# Comorbidity Profiles at Diagnosis in Rheumatoid Arthritis Versus Psoriatic Arthritis: A Nationwide Polish Claims-Based Study

**DOI:** 10.3390/jcm15135249

**Published:** 2026-07-05

**Authors:** Wojciech Zaręba, Mateusz Szeląg, Krzysztof Batko, Piotr Krawiec, Marcin Stajszczyk, Krzysztof Podwójcic, Zbigniew Żuber, Magdalena Krajewska-Włodarczyk, Bogdan Batko

**Affiliations:** 1 Department of Cardiology, J. Dietl Specialist Hospital, Skarbowa 1 St., 31-121 Krakow, Poland; 2 Department of Analysis and Strategy, Ministry of Health, Miodowa 15 St., 00-952 Warsaw, Poland; 3 Department of Dermatology and Allergology, University Hospital, Botaniczna 3 St., 31-503 Krakow, Poland; 4 Doctoral School of Medical and Health Sciences, Jagiellonian University, Łazarza 16 St., 31-530 Krakow, Poland; 5 Department of Rheumatology and Immunology, Faculty of Medicine and Health Sciences, Andrzej Frycz Modrzewski Krakow University, Gustawa Herlinga-Grudzińskiego 1 St., 30-705 Krakow, Poland; 6 Department of Rheumatology and Autoimmune Diseases, Silesian Center for Rheumatology, Szpitalna 11 St., 43-450 Ustroń, Poland; 7 Institute of Labour and Social Studies, Bellottiego 3B St., 01–022 Warsaw, Poland; 8 Department of Pediatrics, Faculty of Medicine and Health Sciences, Andrzej Frycz Modrzewski Krakow University, Gustawa Herlinga-Grudzińskiego 1 St., 30-705 Krakow, Poland; 9 Department of Rheumatology, School of Medicine, Collegium Medicum, University of Warmia and Mazury, Żółnierska 18 St., 10-900 Olsztyn, Poland; 10 Department of Rheumatology and Immunology, J. Dietl Specialist Hospital, Skarbowa 1 St., 31-121 Krakow, Poland

**Keywords:** rheumatoid arthritis, psoriatic arthritis, comorbidity, cardiovascular disease, epidemiology, administrative claims data, Poland

## Abstract

**Background**: Nationwide epidemiologic data on comorbidity burden in early rheumatoid arthritis (RA) and psoriatic arthritis (PsA) are limited. We compared coded diagnoses for concurrent disorders in incident RA and PsA based on administrative healthcare data (AHC). **Methods**: This retrospective cohort study used AHCs from the National Health Fund between 2009 and 2021. Using composite proxy definitions for RA and PsA diagnosis (combination of ICD-10 codes and prescription data), we identified all new cases of RA and PsA between 2019 and 2021. We utilized a ten-year lookback window for the accrual of concurrent disorder claims. Age-, sex-, serostatus- and calendar year-adjusted models were considered. Crude, relative and adjusted prevalence estimates were calculated using generalized linear models. **Results**: Using NHF data, we identified 36,285 and 1603 patients with incident RA/PsA, respectively. We estimated the burden of 31 multisystem comorbidities. Most disorders (*N* = 23, 74.2%) were more frequently coded among RA patients, while only liver diseases were significantly more prevalent in PsA. Chronic back pain (+21.2 pp) and osteoarthritis (+18.3 pp) were tied to the greatest absolute differences, likely mirroring medical contact patterns throughout the differential diagnostic process. Hospitalization due to heart failure and stroke, but not myocardial infarction, was more common in RA vs. PsA. **Conclusions**: Newly diagnosed patients with RA and PsA show distinct patterns of healthcare utilization for multiple organ disorders. Early RA may be tied to higher comorbidity rates not fully explained by age and sex, as compared to PsA; further studies are necessary to clarify these observations.

## 1. Introduction

Rheumatoid arthritis (RA) and psoriatic arthritis (PsA) are systemic, autoimmune arthropathies that are tied to high morbidity and socioeconomic costs [1,2,3,4]. While synovitis is a common feature, the manifestations of RA and PsA are manifold, often affecting different organs (e.g., skin, lung, heart, kidney, eye and endocrine system) [5,6]. Over time, research has shifted from single disease-centered reports to the study of comorbidity patterns and clusters [7,8,9,10,11].

Comorbidity burden arises not only from natural aging, but also develops due to underlying arthritis severity, inflammation and treatment-related factors (e.g., cumulative glucocorticoid (GC) exposure, time to achievement of disease control, length of remission). Cardiovascular (CV), kidney, bone, lung and psychiatric conditions are common concurrent disorders in inflammatory arthritis [10,12,13,14]. Metabolic pathways are regarded as a canonical feature shaping PsA comorbidity. Dysfunctional adiposity and its link to CV, renal and endocrine abnormalities is increasingly recognized as a unifying feature [15,16]. Consensus criteria for metabolic syndrome require at least three of five components (or pharmacologic equivalent): elevated waist circumference, elevated triglycerides, reduced HDL cholesterol, elevated blood pressure, or fasting glucose [17]. Interplay between pathological metabolic and inflammatory pathways appears increasingly relevant for the study of arthritis manifestations [18,19,20,21].

CV disease remains a major cause of excess morbidity and mortality in both RA and PsA, though with variability in predisposing risk factors [18,22,23,24,25]. Systemic inflammation is considered a major driver of abnormal adipocyte function and vascular remodeling, which carries implications for our interpretation of extra-articular effects of disease-modifying antirheumatic drugs (DMARDs), non-steroidal anti-inflammatory agents (NSAIDs) and GCs. Relatively common scenarios in management of RA patients include paradoxical associations between lipid abnormalities and enhanced CV risk [18,26,27]. In turn, CV risk in PsA is more centrally associated with excess adipose tissue and glucose metabolism impairment, which is thought to synergistically affect inflammatory articular pathways [18,28].

Data directly comparing comorbidities in incident RA and PsA are limited [29,30]. Meanwhile, the natural history of RA and PsA is changing with introduction of new therapeutic agents and stringent inflammation control strategies [31]. Current evidence on comorbidity in the literature is derived from studies in established RA and PsA, in which risk is shaped by arthritis severity, conventional synthetic (cs-) and biologic (b-) DMARD exposure, as well as cumulative GC use [11,21,22,32]. Delays in diagnosis of arthritis and DMARD initiation further confound our understanding of risk association between arthritis and specific comorbidities. In PsA particularly, heterogeneous manifestations and lack of specific biochemical/serological tests may further delay diagnosis [33,34,35]. Therefore, studies that report comorbidity profiles at RA/PsA onset are informative; further, quantifying and describing multimorbidity patterns preceding arthritis diagnosis may support earlier recognition strategies [36,37,38].

We conducted a nationwide, retrospective longitudinal study using administrative healthcare claims (AHCs). The primary objective was to describe and compare an array of concurrent disorders based on claims data across RA and PsA, with a unique feature of a ten-year accrual window. We used a pre-defined set of multiorgan disorders that are also common in the general population and/or are plausibly tied to inflammatory arthritis pathways, in line with earlier work by England et al. and Crowson et al. [10,39]. Although AHCs have several limitations, our previously derived epidemiologic estimates were consistent with population-based studies, which provides preliminary construct validity for our approach [40,41,42].

## 2. Materials and Methods

### 2.1. Data Source, Policy and Processing

We sourced AHC data from the Polish Narodowy Fundusz Zdrowia (otherwise referred to as National Health Fund; NHF). The study window for data extraction lasted from 1 January 2009 to 31 December 2021. At present, the NHF is the sole payer of public medical services in Poland, which provides very high population coverage. Healthcare services are provided freely for all citizens with confirmed employment status, though care is also granted in other circumstances (e.g., acute scenarios, pediatric patients). Primary and specialist outpatient centers, as well as hospitals across reference levels, are reimbursed for services based on diagnostic claims under specific regulatory requirements. We are able to conduct a nation-level analysis due to linkage of medical reimbursement records to a unique citizen identifier (PESEL), which is similar to social security/insurance numbers used in other countries. Due to data policy, we are not able to access patient-level data and are only able to examine de-identified, aggregate samples. Ethics committee approval is waived due to conducting retrospective analyses on anonymized records.

During the preparation of this manuscript, the authors used Claude Opus 4.8 (Anthropic) for the purposes of English language corrections, text revision and code development and/or review. The authors have reviewed and edited the output and take full responsibility for the content of this publication.

### 2.2. Study Population

We identified patients with RA and PsA based on a pre-defined case definition, in keeping with earlier work [40,41,42]. RA and PsA are referred to as index conditions; we established diagnoses using an operational definition based on AHCs. We required repeat coding of eligible claims under specific ICD-10 diagnostic codes that occurred at least 90 days apart, together with evidence for arthritis therapy based on centralized pharmaceutical prescription records [40,41]. We only included persons of at least 16 years of age in this analysis. Due to the use of a temporal definition, we defined the index date (corresponding to proxy RA/PsA diagnosis date) as the date on which both algorithm elements were first satisfied. Repeat (confirmatory) claims could be registered for a maximum of 12 months after the first diagnostic code; the index date may fall after the date of the first recorded diagnosis. The use of a ten-year lookback window for accrual of comorbidities was determined with respect to the index date; concurrent disorders are likely to represent the burden around the time of arthritis identification.

RA was defined as repeat diagnostic claims from any level of healthcare contact, coded as M05/M06 at least 90 days apart, together with at least one reimbursed prescription for a csDMARD (methotrexate, sulfasalazine, or leflunomide). Alternatively, the latter could be substituted by patient participation in an NHF-funded b- or targeted synthetic (ts-) DMARD drug program.

PsA was defined using analogous criteria, with the requirement of at least two records of healthcare contact coded as M07.0-M07.3 or L40.5 at least 90 days apart, together with at least one reimbursed prescription for a csDMARD (methotrexate, leflunomide, sulfasalazine, ciclosporin), an NSAID, or a b- or tsDMARD (TNF inhibitor, IL-17 inhibitor, or tofacitinib). Agents not reimbursed during the study period (guselkumab, risankizumab, bimekizumab, ustekinumab, upadacitinib) were excluded from the treatment criterion.

Treatment criteria within this study are asymmetrical in terms of csDMARD inclusion, which is a deliberate choice. Real-world prescribing patterns differ between RA and PsA [43,44,45]. An estimated 90% of patients with RA are treated with methotrexate in Poland [46]. Methotrexate is the anchor drug for RA management; therefore, requiring a DMARD is unlikely to reduce sensitivity for true RA diagnosis. In turn, NSAID monotherapy remains a first-line choice for mild, oligoarticular or axial PsA. Therefore, requiring csDMARDs/biologics for PsA would introduce greater selection bias towards severe, difficult-to-manage arthritis. We did not test alternative, narrower PsA definitions due to the likelihood of lowering sensitivity, though we did perform stratified analyses of RA seropositivity (see Supplementary Table S4).

### 2.3. Definitions

Concurrent disorders were determined using NHF claims data based on a pre-defined set of ICD-10 codes (Supplementary Table S1). For chronic diseases, we set the requirement of at least two relevant records recorded at any level of medical service (e.g., outpatient, inpatient, or rehabilitation claims). Additionally, we included only diagnoses recorded with eligible ICD-10 codes within the pre-specified timeframe, which carries the possibility of missed diagnoses if patients did not experience any public healthcare contact. For acute CV hospitalization outcomes, we identified events based on single inpatient discharge diagnosis codes. The pre-set exclusion criteria are a complementary element of the algorithmic definition; patients were not classified as incident arthritis cases if: (i) only a singular eligible code was recorded, (ii) they lacked antirheumatic therapy prescription evidence, (iii) were recorded with a qualifying diagnosis during the 2009–2018 washout period, (iv) or if they were younger than 16 years. In the NHF, ICD-10 codes are registered by physicians as part of the prerequisite visit elements, which is a reimbursement requirement; diagnostic codes are not encoded by administrative personnel. Therefore, the requirement for at least two physician diagnostic codes, at least 90 days apart, in combination with disease-specific prescriptions, shapes the backbone of the operational arthritis definition. Due to anonymity, we are unable to validate the codes at the patient level.

### 2.4. Statistical Analysis

We performed analysis in R (version 4.5.3; R Core Team, 2026; R Foundation for Statistical Computing, Vienna, Austria) using the publicly available packages (brglm2, MASS, readxl, janitor and tidyverse).

For each ICD-10 disorder group, we derived estimates of crude prevalence and 95% confidence intervals (CIs) using the Wilson score approach. For RA versus PsA differences, we used contrasts that were defined as prevalence differences (percentage points [pp]) and prevalence ratios (PRs) with 95% CIs based on Haldane–Anscombe or log-based approach. Additionally, to account for zero event rates, we predicated our analyses on the assumption that a correction of 0.5 was applied to any zero cell count or cell equal to stratum total. Thereafter, we used chi-square test or Fisher’s exact test, as appropriate and dependent on cell counts.

In order to adjust estimates, we used a binomial generalized linear model with a logit link for each disorder using aggregated age group and calendar year stratum-level counts as the response variable. Models were constructed with arthritis group (RA vs. PsA), age at diagnosis (16–44 vs. 45–100 years) group, and calendar year of diagnosis (2019–2021) group. We used bias-reduced estimation via brglm2 as the main approach; if models did not converge or failed, standard maximum likelihood was used. For adjusted prevalence, we obtained estimates by weighting stratum-level predictions for both RA and PsA. CIs for standardized risk association measures were derived using parametric bootstrap with 5000 iterations.

To correct for false discovery, we used the Benjamini–Hochberg approach with two different family groups: (i) chronic disorders and (ii) acute CV hospitalization events. We excluded sex-specific conditions (e.g., prostatic hyperplasia, non-inflammatory gynecological disease) and dermatologic conditions due to construct bias.

For sensitivity analyses, we calculated sex-, age- and disease subtype-specific model estimates (see Supplementary Tables S4–S6). Due to the aggregate nature of anonymous administrative data, a small-cell disclosure of at least three cases was maintained. Therefore, strata for coded diagnoses below this threshold are treated as zero. Due to the low event count/small-cell rule, we report the stratum coverage, as estimates for strata may not truly be zero. Fully adjusting models for age group, sex and disease subtype is infeasible due to count considerations.

Tests were two-tailed and we treated *p* < 0.05 as statistically significant.

## 3. Results

### 3.1. Study Cohort Characteristics

Using the adopted case definitions, we identified 36,285 patients aged ≥16 years with incident RA and 1603 aged ≥16 years with incident PsA based on records of the Polish NHF between 1 January 2019 and 31 December 2021. Basic group characteristics are shown in Table 1. As expected, the RA cohort sample was substantially greater than the PsA cohort, which is in line with prior Polish epidemiologic estimates.

We derived stable age- and calendar year of diagnosis-adjusted estimates based on binomial models for 31 conditions. Due to differences in incident RA and PsA samples, there were several low-frequency conditions with near-zero or zero event rates, which limited our ability to use the same modeling structure (crude estimates are reported separately in Supplementary Table S2).

### 3.2. Burden of Concurrent Disorders in RA Versus PsA

We analyzed ICD-10 diagnostic codes for which at least three cases were identified. We followed a multiorgan comorbidity structure of 31 conditions, for which stable adjusted risk association measures were estimated. Of the latter, 23 (74.2%) were significantly more prevalent in RA than in PsA after BH correction (Table 2). Only liver disorders were significantly more prevalent in PsA, while seven disorders showed comparable rates across RA and PsA groups.

We observed the largest absolute adjusted differences for musculoskeletal and neurological conditions, as expected. ICD-10 codes for chronic back pain were the most common concurrent diagnosis in RA (44.1% vs. 22.9% in PsA; aRD +21.2 pp, 95% CI 18.8–23.5; aPR 1.93, 95% CI 1.74–2.13), followed by osteoarthritis (39.8% vs. 21.5%; aRD +18.3 pp, 95% CI 15.8–20.7; aPR 1.85, 95% CI 1.66–2.07), peripheral nervous system disorders (31.6% vs. 14.0%; aRD +17.6 pp, 95% CI 15.5–19.4; aPR 2.25, 95% CI 1.97–2.58), and severe ophthalmic disease/visual disorders (19.7% vs. 9.5%; aRD +10.2 pp, 95% CI 8.3–11.9; aPR 2.08, 95% CI 1.73–2.51).

The largest relative differences in RA, despite low absolute prevalence in PsA, were observed for gout (aPR 5.94, 95% CI 2.04–16.41), osteoporosis (aPR 5.03, 95% CI 2.47–10.18), and kidney disease (aPR 3.67, 95% CI 1.72–7.91).

Among CV conditions, standardized prevalence was higher in RA for hypertension (17.6% vs. 12.3%; aPR 1.43, 95% CI 1.23–1.69), ischemic heart disease (10.9% vs. 5.8%; aPR 1.87, 95% CI 1.46–2.40), arrhythmia/conduction disorders (9.0% vs. 6.7%; aPR 1.34, 95% CI 1.08–1.65), cerebrovascular disease (4.4% vs. 1.8%; aPR 2.49, 95% CI 1.58–3.93), peripheral vascular disease (4.2% vs. 1.5%; aPR 2.78, 95% CI 1.74–4.54), and valvular heart disease (1.5% vs. 0.5%; aPR 3.20, 95% CI 1.49–6.69). In contrast, liver diseases were more prevalent in PsA (3.8% vs. 2.7%; aRD −1.1 pp, 95% CI −2.4 to −0.2; aPR 0.70, 95% CI 0.53–0.93).

**Figure 1:**
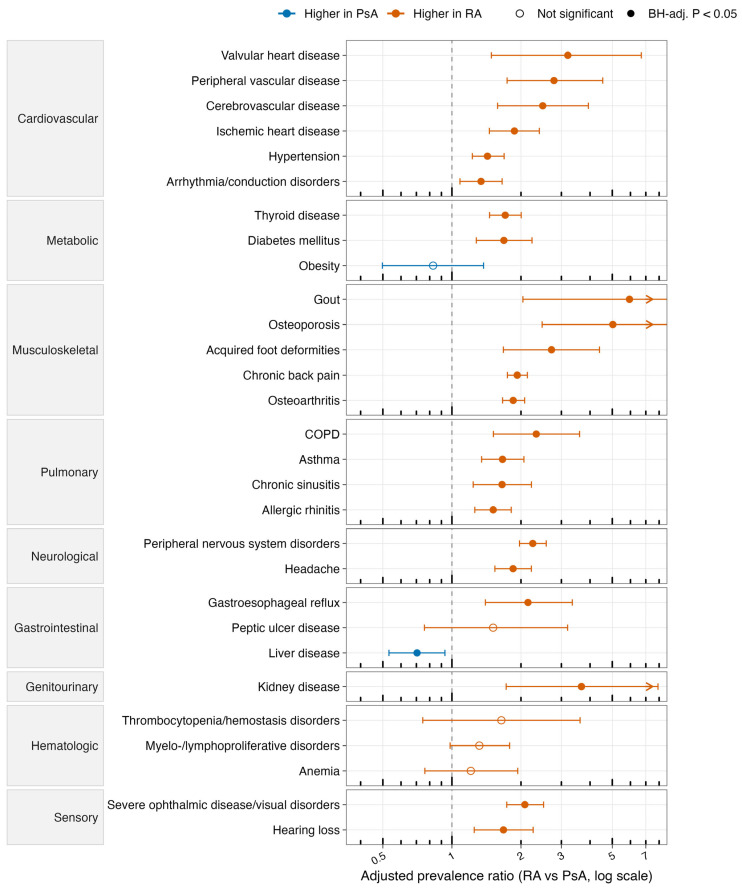
Adjusted prevalence ratios for concurrent disorders in incident RA versus PsA, by organ domain. Forest plot of age- and calendar year-standardized prevalence ratios (RA versus PsA). Error bars are bootstrap-based 95% confidence intervals. Where intervals extended beyond the plotted range, arrows indicate truncation in the direction of the bound.

**Figure 2:**
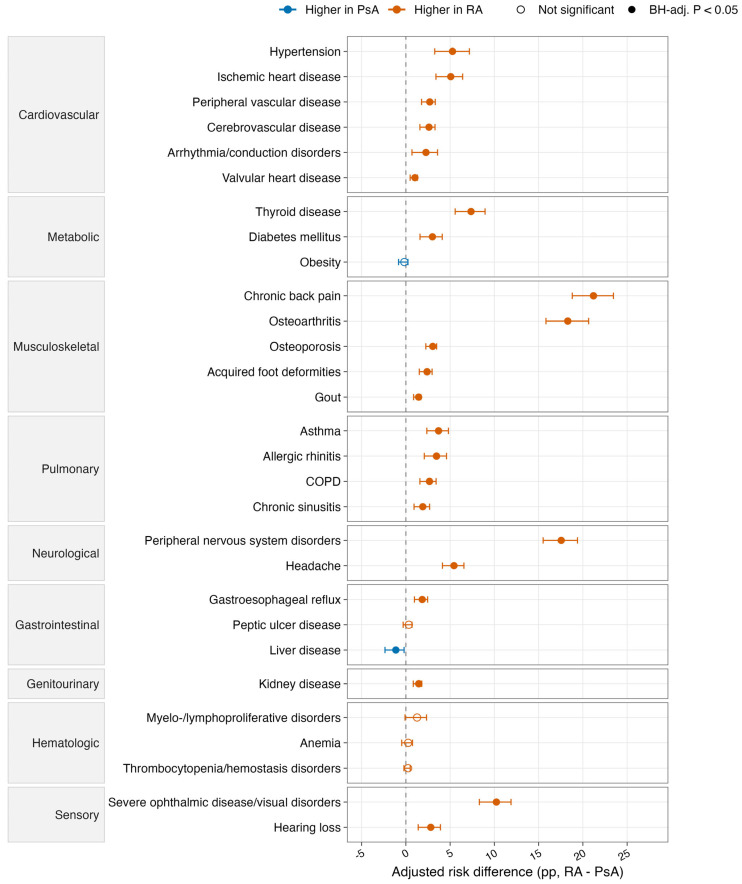
Adjusted risk differences for concurrent disorders in incident RA versus PsA, by organ domain. Forest plot of age- and calendar year-standardized risk differences (absolute difference in prevalence, RA minus PsA, in percentage points [pp]). Error bars are bootstrap-based 95% confidence intervals.

For visual overview, see Figure 1 and Figure 2 for a comparison of adjusted prevalence ratios and risk differences, respectively.

For disorders characterized by low event rates, in which the equivalent adjusted modeling structure was not stable, we provide crude estimates (Supplementary Table S2). In general, for these conditions, the direction is consistently suggestive of greater RA burden, though uncertainty (wide confidence intervals) is very high due to zero or near zero counts in PsA. As an indirect indicator of case definition validity, diagnoses of chronic inflammatory dermatoses were substantially more common in PsA than in RA (58.4% vs. 19.8%; aPR 0.34, 95% CI 0.32–0.36; *p* < 0.001). Due to the intertwined pathophysiology of psoriasis and PsA, this condition was separated from the main analysis structure.

### 3.3. Comorbidity Burden and Age at RA/PsA Diagnosis

Age-stratified results are shown in Supplementary Table S3. Five conditions were consistently more prevalent in RA across both age strata: chronic back pain (aPR 2.12 and 1.95), osteoarthritis (1.77 and 1.94), peripheral nervous system disorders (2.47 and 2.23), thyroid disease (1.96 and 1.61), and headache (1.71 and 1.99). An additional 21 conditions showed excess prevalence in RA only in the older stratum, whereas none were specific to younger patients.

A significant age × cohort interaction was observed for six conditions (Table 2), generally indicating stronger RA excess in older patients. Asthma was not elevated in RA among younger patients (aPR 1.14, BH *p* = 0.462) but was markedly higher in older patients (aPR 2.38, 95% CI 1.69–3.35; BH *p* < 0.001). Similar patterns were observed for hypertension (aPR 0.96 vs. 1.54; BH *p* < 0.001) and hearing loss (aPR 0.72 vs. 2.04; BH *p* < 0.001). In contrast, obesity showed the opposite pattern, with a non-significant increase in younger RA patients (aPR 2.30) and lower prevalence in older RA patients (aPR 0.47, 95% CI 0.28–0.80; BH *p* = 0.015).

### 3.4. Recorded Acute Cardiovascular Hospitalizations in Incident RA Versus PsA

Heart failure hospitalization was more frequent in RA than PsA (784/36,285 vs. 7/791; standardized prevalence 2.5% vs. 0.9%; aPR 2.69, 95% CI: 1.34–5.48; BH *p* = 0.019; Table 3). Stroke showed a similar direction (aPR 2.63, 95% CI: 1.07–6.74) but did not remain significant after BH correction (*p* = 0.060). Myocardial infarction rates were comparable between cohorts (aPR 1.36, 95% CI: 0.81–2.24; BH *p* = 0.245).

Analyses were restricted to PsA patients aged 45–100 years (*n* = 791) due to near-zero event rates in younger patients. RA included both age strata (*n* = 36,285), with standardization for age group and calendar year.

## 4. Discussion

This retrospective nationwide study was based on AHCs sourced from the Polish NHF. Using a ten-year lookback time period, we compared the accrual of diagnostic codes in early RA and PsA. The salient finding of this report is that multiple organ-specific disorders are more commonly recorded in the time prior to or up to RA diagnosis, as compared with PsA. Moreover, hospitalization records for heart failure and stroke are likewise more common in RA, but the rates for myocardial infarction codes are comparable with PsA. Liver conditions are the only disorder group more common in PsA patients. We provide a comprehensive report for multiple comorbidities, with both crude and age-standardized estimates. It is important to consider that crude (unadjusted) prevalence is a better indicator of clinical management decisions at diagnosis, while standardized estimates are more useful for understanding RA/PsA-specific differences.

When comparing comorbidity among incident RA and PsA patients, the difference in age at diagnosis is crucial. In Poland, RA is diagnosed at a median of about 62 years and PsA at about 45 years [40,41]. Estimates from the literature suggest an even broader age range, with RA diagnoses in the sixth and seventh decades [2], which contrast with PsA reports more commonly describing the fourth or fifth decade for diagnosis [35]. The age context is important for interpretation of multimorbidity clustering, particularly when considering its excess. While we observed that age standardization does not fully account for cross-group differences, in favor of excess comorbidity in RA, we were unable to adjust for age as a continuous, quantitative measure (i.e., restriction to analysis of age bands on aggregate data). Studies that adjust for patients’ age describe comparable comorbidity profiles for RA and PsA [47,48,49,50], though only some population studies describe the canonical burden of metabolic disorders in PsA [5,51,52]. In our data, higher rates of liver disorders are the only consistent signal of “classical” metabolic syndrome. Our results are best interpreted as an indirect indicator of early PsA/RA patient risk profiles, including the preclinical stage of arthritis [53,54,55]. The ten-year lookback is also likely to cover for diagnostic delay in inflammatory arthritis diagnosis, which based on previous reports can exceed 1–2 years [33,42].

We observed that several CV disorders (i.e., hypertension, ischemic heart disease, cerebrovascular disease, peripheral vascular disease, and arrhythmia) were more frequently coded for pre-/early RA patients, as were hospitalizations for heart failure (about two-fold increase in risk relative to PsA) and stroke. While suggestive of underlying epidemiologic patterns, these associations are only indirect evidence for enhanced CV risk inherent to RA. Data from the literature document that CV risk is enhanced in both RA and PsA [12,13], though with variable extent and severity. Different underlying pathological pathways are suspected to underlie CV risk profiles in RA (central role of systemic inflammation and altered lipid handling) and PsA (central role of dysfunctional adiposity, insulin resistance, and hepatic steatosis) [18,23,26]. However, population-based studies suggest comparable magnitude of CV risk, when appropriately accounting for traditional risk CV factors [24,56]. At the same time, CV risk evaluation tools perform suboptimally in both RA and PsA [26,28,57,58,59]. Due to the use of secondary-level AHC evidence, we are unable to account for traditional CV risk factors, treatments and disease activity-related factors, therefore our results should not be treated as direct inferential evidence.

The literature describes comparable profiles of CV risk factors between RA and PsA given appropriate adjustment [50]. At present, liver disease was the only condition which was more frequently coded in pre-/early PsA. While theoretically consistent with PsA pathobiology on metabolic dysfunction [19], it may also mirror hepatotoxic effects of csDMARDs or symptom-relief agent (e.g., GCs, NSAIDs) overuse [46]. Although we used an annual population of incident PsA patients, the association with liver disease is not consistent across sex- and seropositivity status. While we observed that standardized rates for obesity are similar, the coded prevalence of obesity (~1%) is unrealistic, likely as a result of underreporting due to social stigma (similar discordance has been observed for mood disorder reporting by physicians in observational studies [22]). At present, due to the lack of real-life validation, it is unknown whether liver disorders are an early, PsA-specific comorbidity [5,60].

We observed that the largest absolute differences (i.e., chronic back pain, osteoarthritis, peripheral nervous system disorders, and severe ophthalmic disease) are most likely reflective of variable diagnostic pathways in inflammatory arthritis, in keeping with the systemic, multiorgan nature of these disorders. Coding practices for “red flag” symptoms and associated differential diagnosis are likely to shape the tractable coded diagnoses across healthcare contacts. Although valuable, claims-based diagnoses are uninformative in terms of the underlying etiology for a large umbrella term, such as chronic back pain; thus, the impact of osteoarthritis-related mechanical/degenerative pathology may not be distinguished from inflammatory nature under the current design. However, the indirect context is consistent with the literature and epidemiology; on average, the older RA patient, with greater concurrent morbidity is more likely to seek medical advice and care. Our results remain consistent with prior work suggesting enhanced risk of osteoporosis [61,62,63], though absolute rates are low. This is partly expected in incident arthritis and is reassuring with respect to pre-diagnostic glucocorticoid exposure. In turn, chronic lung disorders, such as COPD, asthma and chronic sinusitis were also more frequently coded in pre-/early RA. However, in age-stratified analyses, the excess risk of these disorders, and also CV risk factors (e.g., hypertension), was concentrated among older-aged subjects, though interestingly, obesity showed an inverse pattern.

We did not observe evidence for evident RA-PsA differences in hematologic disorders based on the patterns of coded claims. Due to coding practices, we did not examine temporal trends in mental health, which is an important gap for future efforts [64]. The multiorgan association with claims-based diagnoses described in our study are not only an evidence base for descriptive epidemiology, but may be valuable for government bodies and regulatory efforts designing local policies to address multimorbidity. The study of comorbidities in inflammatory arthritis, particularly the profiles recorded at diagnosis, is a crucial aspect for designing of screening, monitoring and management strategies [7,9,39,65,66]. For example, the higher recorded prevalence of inflammatory dermatoses in PsA is consistent with psoriasis preceding arthritis [35,67]; however, psoriasis-related codes also contribute to the PsA case definition, so this is not an independent validation of our design.

Our study has several important strengths and limitations. First, the nationwide scope with high public medical care coverage is a unique scenario that reduces selection bias inherent to single-center studies or insurer/tertiary care-based registry networks. AHCs from the Polish NHF are useful due to data capture from multiple settings that are not usually covered in studies (i.e., all healthcare reference levels, both inpatient and outpatient setting, inclusion of rehabilitation services). We also utilized composite case definitions that combine repeat ICD-10 diagnoses with prescription evidence. In turn, the main limitation is the indirect administrative data source, which is prone to multiple sources of bias (e.g., reimbursement-oriented coding, underreporting). Furthermore, we are unable to obtain patient-level data and source information of disease activity, treatment patterns and other clinically relevant confounders. As such, our interpretation of inflammatory arthritis-derived risk is speculative, and our evidence cannot be treated as inferential. The aggregated, de-identified data structure also limits formal statistical choices. We are unable to utilize patient-level models that would account for established CV risk factors, such as smoking habits, body mass indices, serology, socioeconomic status, and treatment characteristics. For sensitivity, we examined sex and serostatus. Additionally, the operational definitions of RA and PsA may conceivably miss mild disease managed without pharmacotherapy, particularly since the diagnostic (index) date can occur up to 12 months after the first claim. Lastly, the study window overlapped with the COVID-19 pandemic, which may have reduced ascertainment [68,69].

## 5. Conclusions

This was a nationwide healthcare database study from Poland based on National Health Fund records. Using coded reimbursement data for all medical services, we tracked longitudinal patterns of comorbidity. Patients with newly diagnosed RA entered care pathways with higher comorbidity rates than those with newly diagnosed PsA. Although some of these differences are likely explained by the differing age distributions, since RA patients are older, the differences between RA and PsA persisted after adjustment for age group and calendar year of diagnosis. Our findings point to a systemic need to account for the multimorbidity clusters specific to inflammatory arthritis. By extension, they argue for interdisciplinary management, and as a first step we suggest comprehensive evaluation at the time of inflammatory arthritis diagnosis. Given their administrative nature, these data may help inform local policy and provide an important reference point for Central and Eastern Europe. It should be stated explicitly, however, that our results reflect administratively recorded, coded estimates of comorbidity rather than direct clinical assessment at the patient level.

## Figures and Tables

**Table 1:** Baseline characteristics of the incident RA and PsA cohorts, 2019–2021.

Characteristics	RA (*n* = 36,285)			PsA (*n* = 1603)		
	Overall	16–44 y	45–100 y	Overall	16–44 y	45–100 y
Age at diagnosis, years	61 (51–69)	37 (30–41)	64 (56–71)	44 (33–56)	33 (24–39)	56 (49–63)
Female	26,100 (72%)	3910 (71%)	22,190 (72%)	865 (54%)	414 (51%)	451 (57%)
Mortality	646 (1.78%)	4 (0.07%)	642 (2.08%)	10 (0.62%)	3 (0.37%)	7 (0.88%)

Data are presented as median (IQR) or *n* (%), unless otherwise stated. Mortality data were calculated up to 31 December 2021. Mortality is reported for descriptive purposes; the low number of deaths in PsA group and lack of patient-level data preclude time-to-event analyses.

**Table 2:** Prevalence of concurrent disorders after age and calendar year of diagnosis standardization, compared across incident RA and PsA cohorts.

Condition	Std. RA Prevalence (95% CI)	Std. PsA Prevalence (95% CI)	aRD, pp (95% CI)	aPR (95% CI)	BH-Adj. *p*	*p* [Age Int.]
Arrhythmia/conduction disorders	9.0 (8.7–9.3)	6.7 (5.4–8.2)	2.3 (0.7–3.6)	1.34 (1.08–1.65)	0.007	0.328
Cerebrovascular disease	4.4 (4.2–4.6)	1.8 (1.1–2.8)	2.6 (1.6–3.3)	2.49 (1.58–3.93)	<0.001	0.235
Hypertension	17.6 (17.2–18.0)	12.3 (10.4–14.3)	5.3 (3.3–7.2)	1.43 (1.23–1.69)	<0.001	0.022
Ischemic heart disease	10.9 (10.6–11.2)	5.8 (4.5–7.5)	5.1 (3.4–6.4)	1.87 (1.46–2.40)	<0.001	0.388
Peripheral vascular disease	4.2 (4.0–4.4)	1.5 (0.9–2.4)	2.7 (1.8–3.3)	2.78 (1.74–4.54)	<0.001	0.184
Valvular heart disease	1.5 (1.4–1.6)	0.5 (0.2–1.0)	1.0 (0.5–1.3)	3.20 (1.49–6.69)	0.004	0.581
Diabetes mellitus	7.4 (7.1–7.7)	4.4 (3.3–5.8)	3.0 (1.6–4.1)	1.68 (1.28–2.23)	<0.001	0.390
Obesity	0.8 (0.7–0.9)	1.0 (0.6–1.7)	−0.2 (−0.8–0.2)	0.83 (0.50–1.37)	0.478	0.010
Other metabolic disorders	0.1 (0.1–0.2)	0.0 (0.0–0.5)	0.1 (−0.3–0.2)	4.38 (0.28–65.73)	0.323	0.892
Thyroid disease	17.8 (17.4–18.2)	10.4 (8.9–12.1)	7.4 (5.6–8.9)	1.71 (1.46–2.00)	<0.001	0.357
Vitamin excess/hyperalimentation sequelae	0.0 (0.0–0.1)	0.1 (0.0–0.7)	−0.0 (−0.7–0.0)	0.41 (0.03–5.09)	0.486	0.717
Acquired foot deformities	3.8 (3.6–4.0)	1.4 (0.9–2.3)	2.4 (1.5–3.0)	2.72 (1.68–4.40)	<0.001	0.091
Chronic back pain	44.1 (43.6–44.6)	22.9 (20.7–25.3)	21.2 (18.8–23.5)	1.93 (1.74–2.13)	<0.001	0.445
Gout	1.7 (1.6–1.9)	0.3 (0.1–0.8)	1.4 (0.9–1.7)	5.94 (2.04–16.41)	0.001	0.044
Osteoarthritis	39.8 (39.3–40.3)	21.5 (19.2–23.9)	18.3 (15.8–20.7)	1.85 (1.66–2.07)	<0.001	0.033
Osteoporosis	3.8 (3.6–4.0)	0.8 (0.4–1.5)	3.0 (2.3–3.5)	5.03 (2.47–10.18)	<0.001	>0.99
Allergic rhinitis	10.2 (9.9–10.5)	6.7 (5.6–8.1)	3.5 (2.1–4.6)	1.51 (1.26–1.81)	<0.001	0.090
Asthma	9.3 (9.0–9.6)	5.6 (4.5–6.8)	3.7 (2.4–4.8)	1.66 (1.35–2.06)	<0.001	0.001
COPD	4.7 (4.5–4.9)	2.0 (1.3–3.1)	2.7 (1.6–3.4)	2.33 (1.52–3.60)	<0.001	0.841
Chronic sinusitis	4.8 (4.6–5.1)	2.9 (2.2–3.9)	1.9 (0.9–2.7)	1.65 (1.24–2.22)	<0.001	0.174
Headache	11.9 (11.5–12.2)	6.4 (5.3–7.7)	5.4 (4.1–6.6)	1.85 (1.54–2.22)	<0.001	0.492
Peripheral nervous system disorders	31.6 (31.1–32.1)	14.0 (12.3–16.0)	17.6 (15.5–19.4)	2.25 (1.97–2.58)	<0.001	0.912
Gastroesophageal reflux	3.5 (3.3–3.7)	1.6 (1.0–2.4)	1.8 (1.0–2.5)	2.15 (1.40–3.35)	<0.001	0.368
Liver disease	2.7 (2.5–2.9)	3.8 (2.9–5.1)	−1.1 (−2.4–0.2)	0.70 (0.53–0.93)	0.018	0.594
Peptic ulcer disease	1.0 (0.9–1.1)	0.6 (0.3–1.3)	0.3 (−0.3–0.7)	1.51 (0.76–3.19)	0.300	0.294
Kidney disease	2.0 (1.9–2.2)	0.5 (0.3–1.2)	1.5 (0.8–1.8)	3.67 (1.72–7.91)	0.001	0.303
Anemia	1.5 (1.4–1.6)	1.3 (0.8–2.0)	0.3 (−0.5–0.7)	1.21 (0.76–1.94)	0.467	0.407
Myelo-/lymphoproliferative disorders	5.3 (5.0–5.5)	4.0 (2.9–5.3)	1.3 (−0.1–2.3)	1.32 (0.98–1.78)	0.084	0.291
Thrombocytopenia/hemostasis disorders	0.7 (0.6–0.8)	0.4 (0.2–0.9)	0.3 (−0.2–0.5)	1.64 (0.75–3.62)	0.256	0.883
Hearing loss	7.0 (6.7–7.2)	4.2 (3.1–5.6)	2.8 (1.4–3.9)	1.68 (1.25–2.26)	<0.001	0.002
Severe ophthalmic disease/visual disorders	19.7 (19.3–20.1)	9.5 (7.9–11.4)	10.2 (8.3–11.9)	2.08 (1.73–2.51)	<0.001	0.054

Estimates are from binomial GLMs standardized by age group (16–44 and 45–100 years) and calendar year (2019–2021), with 95% parametric-bootstrap CIs. BH correction was applied within comorbidity families. The [age int.] column gives the *p* value for the age × cohort interaction. Std., standardized; aRD, adjusted risk difference; aPR, adjusted prevalence ratio; BH, Benjamini–Hochberg; CI, confidence interval; GLM, generalized linear model; pp, percentage points.

**Table 3:** Recorded acute CV hospitalizations in RA versus PsA.

Condition	RA *n*/*N*	PsA *n*/*N* *	Std. RA Prevalence (95% CI)	Std. PsA Prevalence (95% CI) *	aRD, pp (95% CI)	aPR (95% CI)	*p* Value	BH-Adj. *p* Value
Heart failure hospitalization	784/36,285	7/791	2.5 (2.4–2.7)	0.9 (0.5–1.9)	1.6 (0.6–2.1)	2.69 (1.34–5.48)	0.006	0.019
Stroke hospitalization	471/36,285	4/791	1.5 (1.4–1.6)	0.6 (0.2–1.4)	0.9 (0.1–1.3)	2.63 (1.07–6.74)	0.040	0.060
Myocardial infarction hospitalization	767/36,285	14/791	2.5 (2.3–2.7)	1.8 (1.1–3.1)	0.7 (−0.6–1.4)	1.36 (0.81–2.24)	0.245	0.245

Estimates are from binomial GLMs standardized by age group and calendar year, with 95% parametric-bootstrap CIs; *n*/*N* are crude counts. BH correction was applied within the acute cardiovascular family (*n* = 3). * PsA restricted to ages 45–100 years (*n* = 791) owing to near-zero events below 45. aRD, adjusted risk difference; aPR, adjusted prevalence ratio; BH, Benjamini–Hochberg; CI, confidence interval; GLM, generalized linear model; pp, percentage points.

## Data Availability

In keeping with the previous epidemiologic studies, the data in this study were obtained with the permission of the Ministry of Health of the Republic of Poland from electronic databases of the National Health Fund (NFZ). Datasets are not publicly available because they contain sensitive data at an individual level. Aggregated data may be requested from the Department of Analyses and Strategies in the Ministry of Health in accordance with the provisions on access to public information (a justification for the public interest is required).

## Supplementary Materials

The following supporting information can be downloaded at: https://www.mdpi.com/article/10.3390/jcm15135249/s1, Supplementary Table S1. Dictionary of ICD-10 codes and corresponding claims used to define specific disorders.; Supplementary Table S2. Crude comorbidity prevalence for conditions with unstable adjusted models.; Supplementary Table S3. Age-stratified comorbidity prevalence in RA vs. PsA.; Supplementary Table S4. Sensitivity analyses for relative burden of comorbidities in RA vs. PsA.; Supplementary Table S5. Sex stratified analyses for relative burden of comorbidities in RA vs. PsA.; Supplementary Table S6. Age stratified analyses for all coded comorbidities in rheumatoid arthritis versus psoriatic arthritis.; Figure S1. Age-stratified risk differences for coded concurrent disorders.

## Abbreviations

The following abbreviations are used in this manuscript:

aPR	Adjusted Prevalence Ratio
aRD	Adjusted Risk Difference
BH	Benjamini–Hochberg
b/tsDMARD	Biologic/Targeted Synthetic Disease-Modifying Antirheumatic Drug
CI	Confidence Interval
COPD	Chronic Obstructive Pulmonary Disease
COVID-19	Coronavirus Disease 2019
csDMARD	Conventional Synthetic Disease-Modifying Antirheumatic Drug
DMARD	Disease-Modifying Antirheumatic Drug
FDR	False Discovery Rate
GLM	Generalized Linear Model
ICD-10	International Classification of Diseases, Tenth Revision
IL-17	Interleukin-17
IQR	Interquartile Range
LDL	Low-Density Lipoprotein
NHF	National Health Fund
NSAID	Non-Steroidal Anti-Inflammatory Drug
PESEL	Polish National Identification Number
pp	Percentage Points
PR	Prevalence Ratio
PsA	Psoriatic Arthritis
RA	Rheumatoid Arthritis
TNF	Tumor Necrosis Factor
