# Supplementary material for: Comorbidity Profiles at Diagnosis in Rheumatoid Arthritis Versus Psoriatic Arthritis: A Nationwide Polish Claims-Based Study
Source: J Clin Med. 2026 Jul 5;15(13):5249. doi: 10.3390/jcm15135249 (PMC13363413; doi:10.3390/jcm15135249)
Supplement: Supplementary file 1 [file jcm-15-05249-s001.zip › jcm-4373095-supplementary.pdf]

Table S1. Dictionary of ICD-10 codes and corresponding claims used to define specific disorders.

Disorder	ICD-10 Code(s)	Description
Anemia	D50-D64	All forms of anemia (iron-deficiency, hemolytic, aplastic)
Solid malignant neoplasms	C00-C80	Malignant solid-organ tumors
In situ neoplasms	D00-D09	Carcinoma in situ
Hematological malignancies	C81-C96	Lymphoid and hematopoietic cancers
Myeloid/lymphoproliferative neoplasms of uncertain behavior	D45-D47	Polycythemia vera, myelodysplastic and related disorders
Thrombocytopenia and coagulation/platelet disorders	D65-D69	Bleeding, clotting, and platelet disorders
Leukopenia and leukocyte disorders	D70-D72	Low or abnormal white-cell counts
Cardiac arrhythmias and conduction disorders	I44-I49	Arrhythmias and heart block
Complication of cardiac rhythm device	T82.1	Pacemaker/defibrillator device complications
Cerebrovascular diseases	I60-I69	Stroke and related cerebrovascular disease
Heart failure	I50; I11.0; I13.0; I13.2	Heart failure, including hypertensive forms
Ischemic heart disease	I20-I25	Angina, myocardial infarction, chronic ischemic disease
Arterial hypertension	I10-I15	Essential and secondary hypertension
Valvular heart disease	I05-I08; I34-I39	Rheumatic and non-rheumatic valve disorders
Pulmonary circulatory disorders	I26-I28	Pulmonary embolism and pulmonary hypertension
Peripheral vascular disease	I70-I79	Atherosclerosis and other peripheral arterial disease
Asthma	J45-J46	Asthma and status asthmaticus
COPD and emphysema	J43-J44	Chronic obstructive pulmonary disease and emphysema
Interstitial lung disease	J84	Interstitial and diffuse parenchymal lung disease
Chronic sinusitis	J32	Chronic sinusitis
Allergic/non-infectious rhinitis	J30-J31	Allergic and chronic rhinitis

Diabetes mellitus	E10-E14	Type 1, type 2, and other diabetes
Dyslipidemia	E78	Lipid metabolism disorders
Obesity	E66	Obesity
Thyroid disorders	E02-E07; E89.0	Hypo-/hyperthyroidism and other thyroid disease
Calcium-phosphate/parathyroid disorders	E20-E21; E83	Parathyroid and mineral-metabolism disorders
Purine and pyrimidine metabolism disorders	E79	Hyperuricemia and related metabolic disorders
Amyloidosis	E85	Amyloidosis
Other metabolic disorders	E88	Other or unspecified metabolic disorders
Vitamin excess/overnutrition effects	E67-E68	Hyperalimentation and vitamin-excess sequelae
Diverticular disease	K57	Diverticulosis and diverticulitis
Gastro-esophageal reflux disease	K21	Gastro-esophageal reflux disease
Peptic ulcer disease	K25-K28	Gastric and duodenal ulcers
Chronic liver disease and complications	B18; I85; K70-K77	Chronic hepatitis, cirrhosis, varices, and complications
Status post liver transplantation	Z94.4	Liver transplant status
Kidney diseases	I12-I13; N03-N19; N25	Chronic kidney disease and related nephropathies
Renal replacement therapy/kidney transplant status	Z49; Z94.0; Z99.2	Dialysis or kidney-transplant status
Benign prostatic hyperplasia	N40	Benign prostatic hyperplasia
Urinary incontinence	R32; N39.3; N39.4	Urinary incontinence
Benign/non-inflammatory gynecological disorders	N80-N95	Endometriosis, prolapse, and other gynecologic disorders
Headache syndromes	G43; G44; R51	Migraine and other headache disorders
Peripheral neuropathies and nerve disorders	G50-G64	Mono- and polyneuropathies
Parkinsonism	G20-G21	Parkinson's disease and secondary parkinsonism
Movement disorders	G24-G26	Dystonia and other movement disorders

Chronic back/spinal disorders	M46-M54	Back and spinal disorders (mechanical, degenerative, inflammatory)
Gout	M10	Gout
Osteoarthritis	M15-M19	Osteoarthritis
Osteoporosis	M80-M82	Osteoporosis, with or without fracture
Acquired foot deformities	M20	Acquired deformities of toes and feet
Chronic inflammatory/autoimmune dermatoses	L10-L14; L20-L30; L40-L45; L50-L54; L71; L93	Psoriasis, dermatitis/eczema, and related inflammatory skin disease
Chronic skin ulcers	L97	Chronic skin and lower-limb ulcers
Hearing loss	H90-H91	Conductive and sensorineural hearing loss
Serious ophthalmic diseases/visual impairment	H16; H25-H28; H30-H35; H40-H42; H47; H54	Cataract, glaucoma, retinal disease, and visual impairment

Supplementary Table S2. Crude comorbidity prevalence for conditions with unstable adjusted models.

Condition	RA prevalence (95% CI)	PsA prevalence (95% CI)	Crude RD, pp (95% CI)	Crude PR (95% CI)	Crude p
Heart failure	2.2 (2.1-2.4)	0.6 (0.3-1.1)	1.7 (1.1-2.0)	3.95 (2.05-7.61)	<0.001
Pulmonary circulation disorders	0.3 (0.3-0.4)	0.0 (0.0-0.2)	0.3 (0.1-0.4)	9.77 (0.61-157.10)	0.016
Calcium-phosphate disorders	0.2 (0.1-0.2)	0.2 (0.1-0.5)	-0.0 (-0.4-0.1)	0.90 (0.28-2.86)	0.753
Dyslipidemia	0.9 (0.8-1.0)	0.0 (0.0-0.2)	0.9 (0.6-1.0)	27.72 (1.73-443.91)	<0.001
Purine/pyrimidine metabolism disorders	0.0 (0.0-0.1)	0.0 (0.0-0.2)	0.0 (-0.2-0.1)	0.93 (0.05-15.84)	>0.99
Interstitial lung disease	0.7 (0.6-0.8)	0.0 (0.0-0.2)	0.7 (0.5-0.8)	22.68 (1.42-363.38)	<0.001
Movement disorders	0.8 (0.7-0.9)	0.0 (0.0-0.2)	0.8 (0.5-0.9)	24.53 (1.53-393.05)	<0.001
Parkinson's disease	0.5 (0.4-0.6)	0.0 (0.0-0.2)	0.5 (0.3-0.6)	16.22 (1.01-260.24)	<0.001
Diverticular disease	1.3 (1.1-1.4)	0.0 (0.0-0.2)	1.3 (1.0-1.4)	40.27 (2.52-644.54)	<0.001
Urinary incontinence	1.2 (1.1-1.3)	0.0 (0.0-0.2)	1.2 (1.0-1.3)	39.30 (2.46-629.00)	<0.001
Hematologic malignancy	0.3 (0.2-0.4)	0.0 (0.0-0.2)	0.3 (0.0-0.4)	9.42 (0.59-151.45)	0.025
Leukopenia/WBC disorders	0.5 (0.4-0.5)	0.0 (0.0-0.2)	0.5 (0.2-0.5)	15.16 (0.94-243.29)	<0.001
In situ neoplasms	0.3 (0.2-0.3)	0.0 (0.0-0.2)	0.3 (0.0-0.3)	8.35 (0.52-134.50)	0.035
Chronic skin ulcers	0.6 (0.5-0.7)	0.0 (0.0-0.2)	0.6 (0.4-0.7)	19.58 (1.22-313.93)	<0.001

Supplementary Table S3. Age-stratified comorbidity prevalence in RA vs. PsA.

Condition	Age group	Std. RA prevalence (95% CI)	Std. PsA prevalence (95% CI)	aRD, pp (95% CI)	aPR (95% CI)	BH-adj. p
Arrhythmia/conduction disorders	16-44	4.5 (4.0-5.1)	3.9 (2.8-5.5)	0.6 (-1.1-1.9)	1.16 (0.80-1.67)	0.488
	45-100	9.9 (9.5-10.2)	6.9 (5.3-8.9)	3.0 (0.9-4.6)	1.43 (1.10-1.85)	0.013
Cerebrovascular disease	16-44	0.8 (0.6-1.1)	0.1 (0.0-1.1)	0.7 (-0.3-1.0)	12.29 (0.75-188.80)	0.221
	45-100	5.1 (4.9-5.3)	2.3 (1.5-3.6)	2.8 (1.5-3.7)	2.18 (1.40-3.43)	0.002
Heart failure	16-44	0.0 (0.0-0.0)	0.0 (0.0-0.0)	0.0 (0.0-0.0)	NA	NA
	45-100	2.6 (2.4-2.8)	1.2 (0.6-2.2)	1.4 (0.4-2.0)	2.19 (1.16-4.05)	0.028
Hypertension	16-44	3.0 (2.6-3.5)	3.2 (2.2-4.6)	-0.1 (-1.6-1.0)	0.96 (0.64-1.45)	0.861
	45-100	20.5 (20.0-20.9)	13.3 (11.1-15.9)	7.1 (4.5-9.4)	1.54 (1.28-1.84)	<0.001
Ischemic heart disease	16-44	0.4 (0.3-0.7)	0.1 (0.0-1.0)	0.4 (-0.5-0.6)	6.67 (0.45-120.59)	0.350
	45-100	13.0 (12.6-13.3)	7.1 (5.5-9.1)	5.8 (3.8-7.5)	1.82 (1.42-2.35)	<0.001
Peripheral vascular disease	16-44	1.0 (0.8-1.3)	0.1 (0.0-1.0)	1.0 (0.0-1.3)	16.23 (1.04-277.14)	0.189
	45-100	4.9 (4.6-5.1)	2.1 (1.3-3.3)	2.8 (1.5-3.6)	2.33 (1.46-3.80)	0.002
Pulmonary circulation disorders	16-44	NA	NA	NA	NA	NA
	45-100	0.3 (0.3-0.4)	0.1 (0.0-1.0)	0.3 (-0.7-0.4)	5.53 (0.35-79.55)	0.274
Valvular heart disease	16-44	1.0 (0.8-1.4)	0.4 (0.2-1.3)	0.6 (-0.3-1.0)	2.31 (0.77-6.66)	0.293
	45-100	1.6 (1.5-1.7)	0.4 (0.2-1.2)	1.1 (0.4-1.5)	3.60 (1.29-10.43)	0.028
Calcium-phosphate disorders	16-44	0.0 (0.0-0.0)	0.0 (0.0-0.0)	0.0 (0.0-0.0)	NA	NA
	45-100	0.2 (0.2-0.3)	0.5 (0.2-1.3)	-0.2 (-1.1-0.0)	0.45 (0.16-1.29)	0.187
Diabetes mellitus	16-44	2.1 (1.8-2.6)	0.9 (0.5-1.9)	1.2 (0.2-1.8)	2.27 (1.10-4.68)	0.124
	45-100	8.4 (8.1-8.8)	5.4 (4.0-7.2)	3.1 (1.2-4.5)	1.58 (1.18-2.13)	0.005
Dyslipidemia	16-44	0.3 (0.2-0.5)	0.1 (0.0-1.1)	0.2 (-0.7-0.5)	4.45 (0.28-71.17)	0.407
	45-100	1.0 (0.9-1.1)	0.1 (0.0-1.0)	0.9 (-0.0-1.1)	15.29 (0.99-255.69)	0.082
Obesity	16-44	1.0 (0.8-1.3)	0.4 (0.2-1.2)	0.6 (-0.2-1.0)	2.30 (0.80-6.67)	0.293
	45-100	0.8 (0.7-0.9)	1.7 (1.0-2.8)	-0.9 (-2.0--0.2)	0.47 (0.28-0.80)	0.015
Other metabolic disorders	16-44	0.2 (0.1-0.4)	0.1 (0.0-1.2)	0.1 (-1.0-0.3)	2.36 (0.15-35.18)	0.550
	45-100	0.1 (0.1-0.2)	0.1 (0.0-1.1)	0.1 (-0.9-0.1)	1.94 (0.12-30.37)	0.680

Purine/pyrimidine metabolism disorders	16-44	0.0 (0.0-0.0)	0.0 (0.0-0.0)	0.0 (0.0-0.0)	NA	NA
	45-100	0.0 (0.0-0.1)	0.1 (0.0-1.0)	-0.0 (-0.9-0.0)	0.55 (0.04-8.47)	0.680
Thyroid disease	16-44	11.0 (10.2-11.9)	5.6 (4.2-7.5)	5.4 (3.4-7.1)	1.96 (1.46-2.62)	<0.001
	45-100	19.1 (18.7-19.6)	11.9 (9.8-14.2)	7.2 (4.9-9.3)	1.61 (1.34-1.95)	<0.001
Vitamin excess/hyperalimentation sequelae	16-44	0.0 (0.0-0.0)	0.0 (0.0-0.0)	0.0 (0.0-0.0)	NA	NA
	45-100	0.0 (0.0-0.1)	0.1 (0.0-1.1)	-0.0 (-1.0-0.0)	0.36 (0.03-6.05)	0.524
Acquired foot deformities	16-44	1.5 (1.2-1.8)	0.1 (0.0-1.0)	1.4 (0.5-1.8)	23.21 (1.55-383.53)	0.124
	45-100	4.3 (4.0-4.5)	2.1 (1.3-3.3)	2.2 (0.9-3.0)	2.05 (1.26-3.35)	0.008
Chronic back pain	16-44	25.6 (24.5-26.8)	12.1 (10.0-14.6)	13.5 (10.8-16.0)	2.12 (1.75-2.58)	<0.001
	45-100	47.8 (47.2-48.3)	24.5 (21.5-27.6)	23.2 (20.1-26.3)	1.95 (1.73-2.22)	<0.001
Gout	16-44	0.6 (0.5-0.9)	0.5 (0.2-1.3)	0.2 (-0.6-0.6)	1.41 (0.49-4.05)	0.550
	45-100	2.0 (1.8-2.1)	0.1 (0.0-1.0)	1.9 (0.9-2.1)	31.03 (1.88-521.79)	0.028
Osteoarthritis	16-44	10.1 (9.4-11.0)	5.7 (4.4-7.6)	4.4 (2.4-6.0)	1.77 (1.31-2.35)	<0.001
	45-100	45.7 (45.2-46.3)	23.6 (20.8-26.6)	22.1 (19.1-25.0)	1.94 (1.72-2.20)	<0.001
Osteoporosis	16-44	0.3 (0.2-0.5)	0.1 (0.0-1.0)	0.3 (-0.7-0.5)	4.76 (0.30-77.41)	0.407
	45-100	4.5 (4.3-4.7)	0.9 (0.5-1.9)	3.5 (2.5-4.1)	4.73 (2.32-9.47)	<0.001
Allergic rhinitis	16-44	11.5 (10.7-12.4)	8.8 (7.0-11.0)	2.7 (0.4-4.7)	1.30 (1.04-1.66)	0.124
	45-100	9.9 (9.6-10.3)	5.5 (4.1-7.3)	4.4 (2.6-5.9)	1.81 (1.36-2.44)	<0.001
Asthma	16-44	6.8 (6.2-7.5)	6.0 (4.5-7.8)	0.9 (-1.0-2.5)	1.14 (0.86-1.52)	0.462
	45-100	9.8 (9.4-10.1)	4.1 (2.9-5.8)	5.7 (4.0-6.9)	2.38 (1.69-3.35)	<0.001
COPD	16-44	0.2 (0.1-0.4)	0.1 (0.0-1.0)	0.1 (-0.8-0.3)	3.06 (0.21-47.21)	0.488
	45-100	5.6 (5.3-5.8)	2.5 (1.6-3.8)	3.1 (1.7-4.0)	2.26 (1.45-3.52)	<0.001
Chronic sinusitis	16-44	5.0 (4.4-5.6)	3.6 (2.6-5.2)	1.3 (-0.3-2.6)	1.37 (0.94-1.99)	0.267
	45-100	4.8 (4.6-5.1)	2.4 (1.5-3.7)	2.5 (1.1-3.3)	2.04 (1.29-3.22)	0.005
Interstitial lung disease	16-44	0.2 (0.1-0.4)	0.1 (0.0-1.0)	0.1 (-0.8-0.3)	2.36 (0.17-35.88)	0.550
	45-100	0.8 (0.7-0.9)	0.1 (0.0-0.9)	0.7 (-0.1-0.9)	12.86 (0.87-197.90)	0.102
Headache	16-44	14.1 (13.2-15.0)	8.2 (6.5-10.3)	5.8 (3.6-7.8)	1.71 (1.35-2.17)	<0.001
	45-100	11.4 (11.1-11.8)	5.7 (4.3-7.6)	5.7 (3.8-7.1)	1.99 (1.50-2.65)	<0.001
Movement disorders	16-44	0.3 (0.2-0.6)	0.1 (0.0-1.1)	0.3 (-0.7-0.5)	5.36 (0.32-82.72)	0.394

	45-100	0.8 (0.8-1.0)	0.1 (0.0-0.9)	0.8 (-0.0-0.9)	13.33 (0.97-210.85)	0.099
Parkinson's disease	16-44	0.0 (0.0-0.0)	0.0 (0.0-0.0)	0.0 (0.0-0.0)	NA	NA
	45-100	0.6 (0.5-0.7)	0.1 (0.0-1.0)	0.5 (-0.4-0.7)	9.52 (0.61-153.62)	0.150
Peripheral nervous system disorders	16-44	18.5 (17.5-19.5)	7.5 (5.8-9.5)	11.0 (8.7-12.9)	2.47 (1.92-3.18)	<0.001
	45-100	34.2 (33.7-34.7)	15.3 (13.0-17.9)	18.9 (16.2-21.3)	2.23 (1.91-2.65)	<0.001
Diverticular disease	16-44	0.0 (0.0-0.0)	0.0 (0.0-0.0)	0.0 (0.0-0.0)	NA	NA
	45-100	1.5 (1.4-1.6)	0.1 (0.0-1.0)	1.4 (0.4-1.6)	23.34 (1.43-341.69)	0.042
Gastroesophageal reflux	16-44	1.7 (1.4-2.1)	1.1 (0.6-2.1)	0.6 (-0.5-1.3)	1.60 (0.79-3.17)	0.354
	45-100	3.8 (3.6-4.0)	1.6 (0.9-2.8)	2.2 (1.1-2.9)	2.42 (1.39-4.20)	0.004
Liver disease	16-44	1.8 (1.5-2.2)	2.3 (1.5-3.6)	-0.5 (-1.8-0.4)	0.78 (0.48-1.28)	0.432
	45-100	2.9 (2.7-3.1)	4.4 (3.2-6.0)	-1.5 (-3.1--0.3)	0.66 (0.48-0.91)	0.028
Peptic ulcer disease	16-44	0.4 (0.2-0.6)	0.1 (0.0-1.0)	0.3 (-0.6-0.5)	5.48 (0.35-89.53)	0.394
	45-100	1.1 (1.0-1.2)	0.9 (0.5-1.9)	0.2 (-0.8-0.7)	1.18 (0.58-2.47)	0.680
Kidney disease	16-44	0.8 (0.6-1.1)	0.1 (0.0-0.9)	0.7 (-0.1-1.0)	12.69 (0.88-194.35)	0.221
	45-100	2.2 (2.1-2.4)	0.8 (0.4-1.8)	1.4 (0.5-1.9)	2.74 (1.27-5.76)	0.020
Urinary incontinence	16-44	0.3 (0.2-0.5)	0.1 (0.0-1.0)	0.2 (-0.7-0.5)	4.58 (0.28-76.27)	0.407
	45-100	1.4 (1.3-1.5)	0.1 (0.0-0.9)	1.3 (0.4-1.5)	22.14 (1.46-332.84)	0.045
Anemia	16-44	1.1 (0.8-1.4)	0.7 (0.3-1.6)	0.4 (-0.5-0.9)	1.59 (0.66-3.83)	0.407
	45-100	1.6 (1.5-1.8)	1.6 (0.9-2.7)	0.0 (-1.1-0.7)	1.02 (0.59-1.73)	0.946
Hematologic malignancy	16-44	0.2 (0.1-0.4)	0.1 (0.0-1.1)	0.1 (-0.9-0.3)	2.98 (0.18-45.08)	0.488
	45-100	0.3 (0.3-0.4)	0.1 (0.0-1.0)	0.3 (-0.7-0.4)	4.99 (0.31-77.98)	0.301
Leukopenia/WBC disorders	16-44	0.5 (0.3-0.7)	0.1 (0.0-1.0)	0.4 (-0.5-0.7)	7.71 (0.50-123.52)	0.307
	45-100	0.5 (0.4-0.6)	0.1 (0.0-0.9)	0.4 (-0.4-0.5)	7.53 (0.51-110.49)	0.196
Myelo-/lymphoproliferative disorders	16-44	1.0 (0.8-1.3)	0.4 (0.2-1.3)	0.6 (-0.3-1.0)	2.28 (0.78-6.47)	0.293
	45-100	6.1 (5.9-6.4)	5.0 (3.6-6.8)	1.1 (-0.7-2.5)	1.23 (0.90-1.68)	0.236
Thrombocytopenia/hemostasis disorders	16-44	0.7 (0.5-1.0)	0.4 (0.2-1.3)	0.3 (-0.6-0.7)	1.63 (0.54-4.61)	0.463
	45-100	0.6 (0.6-0.7)	0.4 (0.2-1.2)	0.2 (-0.6-0.5)	1.45 (0.51-4.20)	0.535
In situ neoplasms	16-44	0.0 (0.0-0.0)	0.0 (0.0-0.0)	0.0 (0.0-0.0)	NA	NA
	45-100	0.3 (0.3-0.4)	0.1 (0.0-0.9)	0.2 (-0.6-0.3)	4.87 (0.33-76.06)	0.302

Hearing loss	16-44	1.2 (1.0-1.5)	1.7 (1.0-2.9)	-0.5 (-1.7-0.3)	0.72 (0.40-1.30)	0.407
	45-100	8.1 (7.8-8.4)	4.0 (2.8-5.6)	4.1 (2.5-5.3)	2.04 (1.44-2.88)	<0.001
Severe ophthalmic disease/visual disorders	16-44	3.7 (3.2-4.2)	2.4 (1.5-3.7)	1.3 (-0.1-2.3)	1.53 (0.98-2.45)	0.221
	45-100	22.9 (22.4-23.4)	10.3 (8.4-12.6)	12.6 (10.2-14.6)	2.22 (1.81-2.74)	<0.001
Chronic skin ulcers	16-44	0.0 (0.0-0.0)	0.0 (0.0-0.0)	0.0 (0.0-0.0)	NA	NA
	45-100	0.7 (0.6-0.8)	0.1 (0.0-1.0)	0.7 (-0.3-0.8)	11.53 (0.68-174.94)	0.117

Supplementary Table S4. Sensitivity analyses for relative burden of comorbidities in RA vs. PsA.

Condition	PsA strata	Primary aPR (95% CI)	Sex-adjusted aPR (95% CI)	Seropositive RA (M05) aPR (95% CI)
Chronic back pain	18/18	1.93 (1.74-2.13)	1.80 (1.64-1.99)	1.85 (1.67-2.05)
Osteoarthritis	18/18	1.85 (1.66-2.07)	1.73 (1.56-1.93)	1.72 (1.55-1.92)
Hypertension	17/18	1.43 (1.23-1.69)	1.39 (1.18-1.64)	1.33 (1.13-1.57)
Peripheral nervous system disorders	17/18	2.25 (1.97-2.58)	2.14 (1.87-2.45)	2.12 (1.86-2.43)
Severe ophthalmic disease/visual disorders	14/18	2.08 (1.73-2.51)	2.12 (1.76-2.57)	2.12 (1.75-2.58)
Arrhythmia/conduction disorders	13/18	1.34 (1.08-1.65)	1.42 (1.14-1.76)	1.32 (1.06-1.65)
Headache	13/18	1.85 (1.54-2.22)	1.74 (1.44-2.11)	1.84 (1.52-2.22)
Thyroid disease	12/18	1.71 (1.46-2.00)	1.49 (1.28-1.76)	1.72 (1.46-2.02)
Allergic rhinitis	11/18	1.51 (1.26-1.81)	1.49 (1.24-1.80)	1.49 (1.23-1.81)
Diabetes mellitus	11/18	1.68 (1.28-2.23)	1.99 (1.50-2.71)	1.73 (1.31-2.35)
Ischemic heart disease	11/18	1.87 (1.46-2.40)	2.00 (1.56-2.60)	1.82 (1.40-2.34)
Asthma	10/18	1.66 (1.35-2.06)	1.66 (1.33-2.07)	1.71 (1.37-2.15)
Chronic sinusitis	9/18	1.65 (1.24-2.22)	1.73 (1.28-2.30)	1.69 (1.23-2.30)
Liver disease	8/18	0.70 (0.53-0.93)	0.89 (0.65-1.20)	0.83 (0.60-1.14)
Hearing loss	7/18	1.68 (1.25-2.26)	2.29 (1.63-3.23)	2.06 (1.48-2.91)
Myelo-/lymphoproliferative neoplasms	7/18	1.32 (0.98-1.78)	1.69 (1.21-2.41)	1.65 (1.16-2.33)
Cerebrovascular disease	3/18	2.49 (1.58-3.93)	3.32 (1.99-5.55)	3.20 (1.90-5.47)
Peripheral vascular disease	3/18	2.78 (1.74-4.54)	4.35 (2.45-7.72)	3.83 (2.17-6.89)
Anemia	2/18	1.21 (0.76-1.94)	1.75 (0.97-3.11)	1.80 (0.99-3.26)
Gastroesophageal reflux	2/18	2.15 (1.40-3.35)	6.25 (2.95-13.34)	5.68 (2.64-12.35)
Obesity	2/18	0.83 (0.50-1.37)	1.58 (0.76-3.27)	1.33 (0.63-2.84)
Acquired foot deformities	1/18	2.72 (1.68-4.40)	5.92 (2.76-12.64)	6.94 (3.30-15.38)
Osteoporosis	1/18	5.03 (2.47-10.18)	5.67 (2.46-13.04)	—
Peptic ulcer disease	1/18	1.51 (0.76-3.19)	3.12 (1.14-8.98)	—
Leukopenia/WBC disorders	<1/18	—	12.40 (0.80-197.30)	12.79 (0.77-212.39)
Other metabolic disorders	<1/18	4.38 (0.28-65.73)	2.87 (0.18-44.15)	1.85 (0.13-26.05)
Purine/pyrimidine metabolism disorders	<1/18	—	0.20 (0.02-2.07)	—
Thrombocytopenia/haemostatic disorders	<1/18	1.64 (0.75-3.62)	19.77 (1.32-307.15)	20.46 (1.19-311.45)
Vitamin excess/hyperalimentation sequelae	<1/18	0.41 (0.03-5.09)	0.29 (0.03-3.42)	0.40 (0.03-4.92)

COPD	3/18	2.33 (1.52-3.60)	—	—
Calcium-phosphate disorders	<1/18	—	—	—
Chronic skin ulcers	<1/18	—	—	—
Diverticular disease	<1/18	—	—	—
Dyslipidemia	<1/18	—	—	—
Gout	<1/18	5.94 (2.04-16.41)	—	—
Heart failure	<1/18	—	—	—
Hematological malignancies	<1/18	—	—	—
In situ neoplasms	<1/18	—	—	—
Interstitial lung disease	<1/18	—	—	—
Kidney disease	<1/18	3.67 (1.72-7.91)	—	—
Movement disorders	<1/18	—	—	—
Parkinson's disease	<1/18	—	—	—
Pulmonary circulation disorders	<1/18	—	—	—
Urinary incontinence	<1/18	—	—	—
Valvular heart disease	<1/18	3.20 (1.49-6.69)	—	—

PsA strata: age×year×sex strata (of 18) with ≥3 cases; ≥80% (≥15/18) considered reliable. — indicate not estimateable models.

Supplementary Table S5. Sex stratified analyses for relative burden of comorbidities in RA vs. PsA.

Condition	Primary aPR (95% CI)	Female aPR (95% CI)	Male aPR (95% CI)
Chronic back pain	1.93 (1.74-2.13)	1.69 (1.51-1.89)	2.20 (1.83-2.66)
Osteoarthritis	1.85 (1.66-2.07)	1.79 (1.57-2.06)	1.72 (1.42-2.10)
Hypertension	1.43 (1.23-1.69)	1.59 (1.28-1.99)	1.18 (0.93-1.50)
Peripheral nervous system disorders	2.25 (1.97-2.58)	1.91 (1.63-2.24)	2.79 (2.16-3.61)
Severe ophthalmic disease/visual disorders	2.08 (1.73-2.51)	1.99 (1.59-2.49)	2.43 (1.71-3.47)
Arrhythmia/conduction disorders	1.34 (1.08-1.65)	1.21 (0.94-1.57)	1.79 (1.20-2.70)
Headache	1.85 (1.54-2.22)	1.81 (1.45-2.27)	1.55 (1.08-2.24)
Thyroid disease	1.71 (1.46-2.00)	1.41 (1.21-1.66)	2.26 (1.28-3.93)
Allergic rhinitis	1.51 (1.26-1.81)	1.53 (1.22-1.93)	1.51 (1.11-2.06)
Diabetes mellitus	1.68 (1.28-2.23)	2.54 (1.60-3.92)	1.55 (1.06-2.29)
Ischemic heart disease	1.87 (1.46-2.40)	1.98 (1.38-2.83)	—
Asthma	1.66 (1.35-2.06)	1.94 (1.46-2.63)	1.35 (0.95-1.94)
Chronic sinusitis	1.65 (1.24-2.22)	2.03 (1.34-3.09)	1.35 (0.87-2.08)
Liver disease	0.70 (0.53-0.93)	1.07 (0.68-1.68)	0.77 (0.51-1.20)
Hearing loss	1.68 (1.25-2.26)	1.68 (1.14-2.51)	3.59 (2.02-6.54)
Myelo-/lymphoproliferative neoplasms	1.32 (0.98-1.78)	1.66 (1.08-2.57)	1.62 (0.96-2.76)
Cerebrovascular disease	2.49 (1.58-3.93)	2.01 (1.18-3.40)	—
Peripheral vascular disease	2.78 (1.74-4.54)	5.02 (1.99-12.51)	3.46 (1.75-7.05)
Anemia	1.21 (0.76-1.94)	1.14 (0.64-2.03)	12.33 (0.83-190.32)
Gastroesophageal reflux	2.15 (1.40-3.35)	4.08 (1.97-9.05)	29.69 (1.91-493.80)
Obesity	0.83 (0.50-1.37)	1.16 (0.55-2.42)	7.57 (0.47-118.54)
Acquired foot deformities	2.72 (1.68-4.40)	4.76 (2.22-10.38)	16.32 (1.05-258.80)
Osteoporosis	5.03 (2.47-10.18)	5.06 (2.15-11.48)	—
Peptic ulcer disease	1.51 (0.76-3.19)	1.64 (0.58-4.59)	—
Leukopenia/WBC disorders	—	9.97 (0.67-171.67)	—
Other metabolic disorders	4.38 (0.28-65.73)	2.87 (0.19-44.62)	—
Purine/pyrimidine metabolism disorders	—	0.20 (0.02-2.52)	—
Thrombocytopenia/haemostatic disorders	1.64 (0.75-3.62)	13.26 (0.82-195.25)	—
Vitamin excess/hyperalimentation sequelae	0.41 (0.03-5.09)	0.29 (0.02-3.79)	—
COPD	2.33 (1.52-3.60)	—	—

Calcium-phosphate disorders	—	—	—
Chronic skin ulcers	—	—	—
Diverticular disease	—	—	—
Dyslipidemia	—	—	7.72 (0.52-119.68)
Gout	5.94 (2.04-16.41)	—	29.70 (1.81-488.69)
Heart failure	—	—	—
Hematological malignancies	—	—	—
In situ neoplasms	—	—	—
Interstitial lung disease	—	—	—
Kidney disease	3.67 (1.72-7.91)	—	21.58 (1.33-352.93)
Movement disorders	—	—	—
Parkinson's disease	—	—	—
Pulmonary circulation disorders	—	—	—
Urinary incontinence	—	—	—
Valvular heart disease	3.20 (1.49-6.69)	24.53 (1.51-388.63)	13.74 (0.84-219.54)

Coverage and reliability as in Table S4. — indicate not estimateable models.

Supplementary Table S6. Age stratified analyses for all coded comorbidities in rheumatoid arthritis versus psoriatic arthritis.

Condition	16-44 y aPR (95% CI)	45-64 y aPR (95% CI)	≥65 y aPR (95% CI)
Chronic back pain	2.03 (1.67-2.48)	1.81 (1.58-2.09)	1.63 (1.32-2.05)
Osteoarthritis	1.69 (1.27-2.26)	1.76 (1.51-2.06)	1.39 (1.17-1.69)
Hypertension	1.05 (0.69-1.62)	1.16 (0.92-1.45)	1.30 (0.99-1.75)
Peripheral nervous system disorders	2.37 (1.85-3.05)	2.22 (1.85-2.69)	1.81 (1.34-2.51)
Severe ophthalmic disease/visual disorders	2.02 (1.17-3.47)	1.56 (1.15-2.11)	1.51 (1.18-2.00)
Arrhythmia/conduction disorders	1.09 (0.75-1.58)	1.13 (0.81-1.60)	1.46 (0.94-2.35)
Headache	1.46 (1.15-1.85)	2.17 (1.53-3.13)	1.93 (1.06-3.59)
Thyroid disease	1.62 (1.20-2.16)	1.26 (1.01-1.57)	1.53 (1.06-2.26)
Allergic rhinitis	1.25 (0.99-1.58)	1.78 (1.25-2.59)	2.00 (1.10-3.75)
Diabetes mellitus	2.62 (1.18-5.78)	1.50 (0.98-2.26)	1.43 (0.89-2.32)
Ischemic heart disease	3.51 (0.26-51.17)	1.45 (1.03-2.07)	1.49 (1.03-2.16)
Asthma	1.09 (0.82-1.46)	2.03 (1.35-3.02)	5.34 (1.91-15.43)
Chronic sinusitis	1.38 (0.92-2.07)	2.02 (1.18-3.47)	2.41 (0.86-6.95)
Liver disease	0.97 (0.58-1.63)	0.63 (0.43-0.94)	10.80 (0.71-183.41)
Hearing loss	0.88 (0.48-1.62)	1.75 (1.06-2.88)	3.00 (1.51-5.89)
Myelo-/lymphoproliferative neoplasms	14.27 (0.91-222.51)	1.02 (0.67-1.56)	1.36 (0.78-2.41)
Cerebrovascular disease	9.61 (0.61-144.89)	1.97 (1.03-3.90)	2.43 (1.10-5.53)
Peripheral vascular disease	14.26 (0.93-237.87)	2.32 (1.14-4.76)	3.12 (1.26-7.87)
Anemia	1.21 (0.50-2.84)	1.20 (0.56-2.57)	6.65 (0.42-111.95)
Gastroesophageal reflux	3.66 (1.29-10.53)	34.04 (2.19-569.17)	2.26 (0.83-6.18)
Obesity	14.07 (0.94-219.35)	0.72 (0.35-1.53)	1.84 (0.12-28.31)
Acquired foot deformities	19.77 (1.29-293.17)	2.86 (1.35-6.04)	15.98 (1.05-228.93)
Osteoporosis	3.23 (0.21-40.99)	19.91 (1.26-336.22)	2.06 (0.91-4.68)
Peptic ulcer disease	4.14 (0.28-57.69)	1.60 (0.54-4.45)	4.91 (0.32-76.17)
Leukopenia/WBC disorders	5.14 (0.33-83.91)	4.54 (0.30-68.90)	1.72 (0.12-27.96)
Other metabolic disorders	1.53 (0.12-20.38)	0.94 (0.07-12.97)	0.35 (0.03-5.12)
Purine/pyrimidine metabolism disorders	—	0.23 (0.02-2.86)	—
Thrombocytopenia/hemostatic disorders	7.66 (0.51-115.75)	7.01 (0.43-120.47)	2.54 (0.18-44.05)
Vitamin excess/hyperalimentation sequelae	—	—	0.15 (0.01-2.05)
COPD	—	4.99 (1.98-12.72)	2.04 (1.06-4.20)

Calcium-phosphate disorders	—	1.12 (0.08-17.70)	0.73 (0.05-10.61)
Chronic skin ulcers	—	5.74 (0.40-89.21)	3.65 (0.28-59.31)
Diverticular disease	—	6.63 (0.46-115.86)	8.20 (0.55-137.07)
Dyslipidemia	—	8.52 (0.53-129.46)	4.16 (0.28-67.23)
Gout	11.97 (0.76-180.98)	23.95 (1.35-366.57)	8.72 (0.59-145.81)
Heart failure	—	14.24 (0.84-220.61)	16.75 (1.06-256.84)
Hematological malignancies	1.06 (0.09-12.41)	2.42 (0.15-37.38)	1.44 (0.09-24.35)
In situ neoplasms	—	1.91 (0.13-29.60)	1.29 (0.09-21.11)
Interstitial lung disease	—	6.95 (0.45-110.19)	4.34 (0.27-70.59)
Kidney disease	12.19 (0.88-179.37)	15.66 (1.10-230.13)	12.05 (0.73-191.90)
Movement disorders	4.58 (0.32-70.89)	5.66 (0.36-91.54)	4.42 (0.26-70.41)
Parkinson's disease	—	2.60 (0.16-40.74)	3.92 (0.27-70.15)
Pulmonary circulation disorders	—	2.88 (0.20-47.92)	1.48 (0.09-25.11)
Urinary incontinence	3.28 (0.24-48.45)	10.34 (0.62-168.78)	5.06 (0.34-88.46)
Valvular heart disease	13.28 (0.88-177.78)	11.17 (0.68-175.33)	8.27 (0.53-140.45)

Coverage and reliability as in Table S4. — indicate not estimateable models.

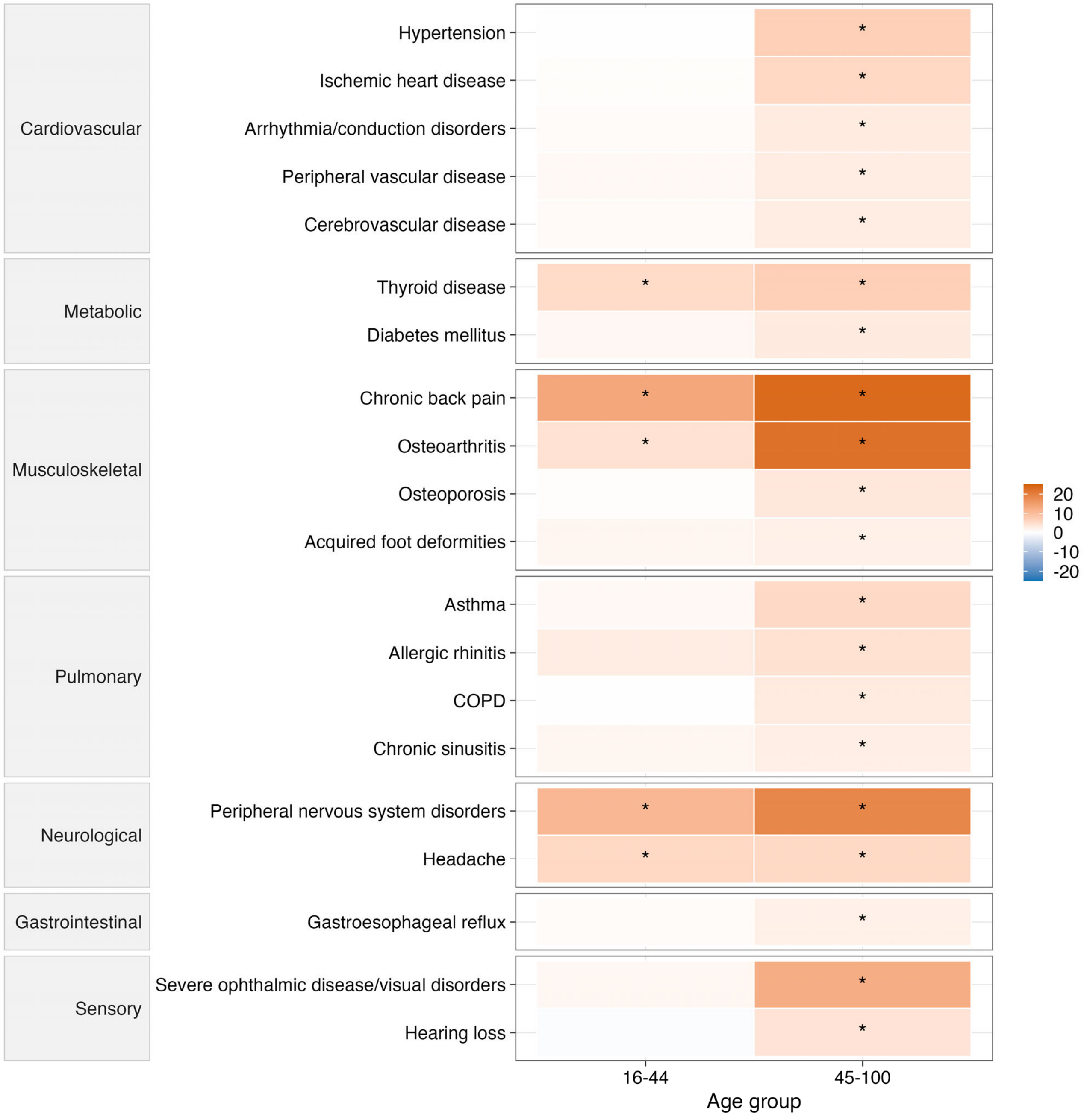
Cardiovascular
Metabolic
Musculoskeletal
Pulmonary
Neurological
Gastrointestinal
Sensory
Hypertension
Ischemic heart disease
Arrhythmia/conduction disorders
Peripheral vascular disease
Cerebrovascular disease
Thyroid disease
Diabetes mellitus
Chronic back pain
Osteoarthritis
Osteoporosis
Acquired foot deformities
Asthma
Allergic rhinitis
COPD
Chronic sinusitis
Peripheral nervous system disorders
Headache
Gastroesophageal reflux
Severe ophthalmic disease/visual disorders
Hearing loss
16-44
45-100
Age group
20
10
0
-10
-20

Figure S1. Age-stratified risk differences for coded concurrent disorders

Heatmap illustration of risk differences for the RA vs. PsA comparison. Conditions that exceed 2 percentage point (pp) thresholds in either age stratum are shown. Orange color corresponds to higher risk in RA, while blue for PsA, respectively. White is indicative of comparable (null) risk estimate. Color scale is based on a symmetric range from -25 to 25 pp. We label significant comparisons by an asterisk (*) based on Benjamini-Hochberg corrected p values < 0.05.
